# Web-Based Privacy-Preserving Multicenter Medical Data Analysis Tools Via Threshold Homomorphic Encryption: Design and Development Study

**DOI:** 10.2196/22555

**Published:** 2020-12-08

**Authors:** Yao Lu, Tianshu Zhou, Yu Tian, Shiqiang Zhu, Jingsong Li

**Affiliations:** 1 Engineering Research Center of EMR and Intelligent Expert System Key Laboratory for Biomedical Engineering of Ministry of Education, College of Biomedical Engineering and Instrument Science Zhejiang University Hangzhou China; 2 Zhejiang Lab Hangzhou China

**Keywords:** machine learning, confidentiality, threshold homomorphic encryption, logistic regression

## Abstract

**Background:**

Data sharing in multicenter medical research can improve the generalizability of research, accelerate progress, enhance collaborations among institutions, and lead to new discoveries from data pooled from multiple sources. Despite these benefits, many medical institutions are unwilling to share their data, as sharing may cause sensitive information to be leaked to researchers, other institutions, and unauthorized users. Great progress has been made in the development of secure machine learning frameworks based on homomorphic encryption in recent years; however, nearly all such frameworks use a single secret key and lack a description of how to securely evaluate the trained model, which makes them impractical for multicenter medical applications.

**Objective:**

The aim of this study is to provide a privacy-preserving machine learning protocol for multiple data providers and researchers (eg, logistic regression). This protocol allows researchers to train models and then evaluate them on medical data from multiple sources while providing privacy protection for both the sensitive data and the learned model.

**Methods:**

We adapted a novel threshold homomorphic encryption scheme to guarantee privacy requirements. We devised new relinearization key generation techniques for greater scalability and multiplicative depth and new model training strategies for simultaneously training multiple models through x-fold cross-validation.

**Results:**

Using a client-server architecture, we evaluated the performance of our protocol. The experimental results demonstrated that, with 10-fold cross-validation, our privacy-preserving logistic regression model training and evaluation over 10 attributes in a data set of 49,152 samples took approximately 7 minutes and 20 minutes, respectively.

**Conclusions:**

We present the first privacy-preserving multiparty logistic regression model training and evaluation protocol based on threshold homomorphic encryption. Our protocol is practical for real-world use and may promote multicenter medical research to some extent.

## Introduction

### Background

In recent years, researchers have proposed strong requirements for the quality of medical research as it continues to progress, which has promoted the development of multicenter research. Compared with single-center research, multicenter research has many significant advantages, including enabling specific analyses for which no single institution has sufficient data, such as on a rare disease; providing medical data from different locations with diverse demographics, which increases the reproducibility and generalizability of the research; and generating pooled medical data that enables new discoveries that cannot be elucidated from any individual data set [1,2]. In addition, the development of multicenter medical research has accelerated the translation of research outcomes into clinical practice and strengthened collaborations among institutions [2,3].

However, data sharing during multicenter research may increase privacy security risks. As medical data are highly sensitive, the leakage of sensitive information will lead to severe consequences, such as financial loss, social discrimination, and unauthorized data abuse, which can harm both patients and medical institutions [4]. As a result, many medical institutions are unwilling to share their data despite the aforementioned benefits, which hinders the collaborative benefits of multicenter research. To solve this problem, a framework is urgently needed to support multicenter medical research efficiently while preventing the leakage of sensitive information.

### Prior Work

Logistic regression is a widely used machine learning approach in various medical applications, such as prognostic prediction, disease diagnosis, and decision-making support [5]. For example, Abdolmaleki et al [6] used logistic regression to predict the outcome of biopsy in breast cancer and obtained 90% accuracy. Many solutions have been developed to address privacy-preserving logistic regression. Some use intermediary statistics to train a model without accessing the raw data; however, these methods remain vulnerable to statistical attack when a particular criterion holds true for only one sample [7-9]. Other researchers use homomorphic encryption to protect privacy during model training, which is similar to that used in this study [10-19]. Homomorphic encryption technology provides rigorous protection for sensitive information and enables the computation of information in an encrypted format and is, therefore, a potential candidate for secure logistic regression model training. However, unlike our solution, these homomorphic encryption–based solutions yield only sets of parameters, and there are no methods to evaluate the trained model in a secure manner. Furthermore, these methods use a single public and secret key, meaning that all the research data may be exposed to anyone who holds the secret key, limiting the application of these solutions in real-life scenarios. In the current literature, the works most similar to ours are those of Emam et al [18] and Jiang et al [19], which attempt to avoid information leak using methods that differ from ours. Emam et al [18] kept the data local to the corresponding data providers and used the Paillier scheme to deal with intermediate values. However, because the public and secret keys are stored at the central unit, when multiple parties collude with the central unit, some meaningful information about the other parties’ sensitive data may be revealed to them [18]. Jiang et al [19] proposed a hybrid cryptographic method that uses a software guard extensions (SGX) enclave to securely generate and store the secret key in a trusted cloud. As the cloud server is shared among different users, it is more likely to be attacked. Considering the rapid development of attack methods toward SGX, including a recently proposed method capable of stealing the enclave secret to subvert the confidentiality of SGX, placement of the secret key in the cloud is not secure [20]. Once the attackers break through the SGX’s guard, they will be able to obtain the secret key and decrypt all the sensitive information stored on the cloud, leading to a severe outcome.

Multikey homomorphic encryption, first proposed by López-Alt et al [21], allows computations on ciphertexts under different secret keys, which makes the method suitable for secure multicenter research. However, the scheme proposed in the study by Lopez et al [21] is based on the Nth degree truncated polynomial ring units cryptosystem, where if we obtain a result computed from ciphertexts under different keys, we will need to decrypt the result by the product of all involved secret keys, allowing for only a very limited number of parties before the decryption error grows too large to obtain the correct plaintext result. Another multikey homomorphic encryption method, called threshold homomorphic encryption, allows many more parties to participate without resulting in an excessively large decryption error; however, the noise generated in the relinearization is still very large and grows quadratically with the number of parties, which would have a negative effect on the multiplicative depth [22].

### Objectives

In this study, we propose a privacy-preserving multicenter research protocol using secure logistic regression, consisting of 3 primary entities: researchers, a service provider, and data providers, in which medical data are horizontally distributed. Our proposed protocol supports not only model training but also the evaluation of the trained model in a secure manner. The protocol guarantees the privacy of both the sensitive data for the data providers and the trained model for the researchers during model training and trained model evaluation. To satisfy privacy requirements, we apply threshold homomorphic encryption and propose a new relinearization key generation process that increases scalability and multiplicative depth. The proposed protocol has been implemented and tested with simulated real-life scenarios. The experimental results demonstrate that our protocol is efficient and practical for real-world applications.

## Methods

### Overview of the Presented Protocol

**Figure 1:**
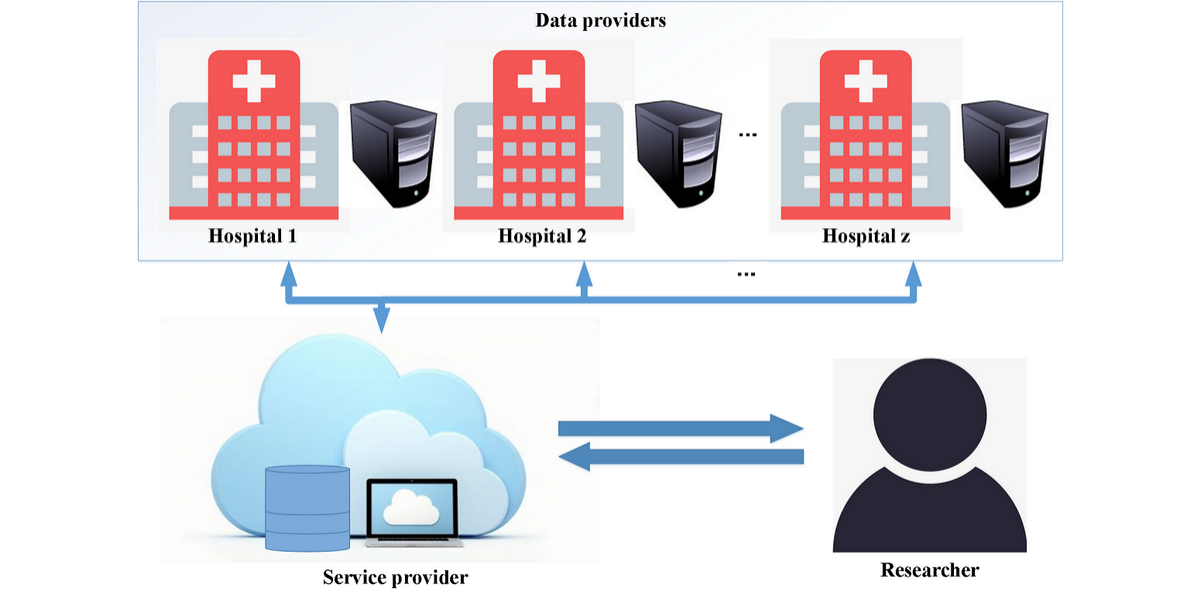
The architecture of the proposed protocol, containing 3 entities: data providers, a service provider, and researchers.

Our proposed protocol includes 3 primary entities as shown below. The architecture of the proposed protocol is shown in Figure 1.

#### Data Providers

These include institutions (eg, hospitals) who hold medical data and are willing to provide these data to the service provider for public use so long as the privacy of the data is preserved. To share medical data, the data providers must obtain patient consent if the local law requires so. Upon receiving the researchers’ requests from the service provider, the data providers can decide whether to accept or refuse. To allow researchers to obtain correct research data, all data providers must implement data standardization to transform the data into a common format, such as the Observational Medical Outcomes Partnership common data model from the Observational Health Data Sciences and Informatics collaborative [23].

#### Service Provider

This refers to an entity that (1) provides storage for encrypted data and research information, (2) performs the most computationally expensive part of the privacy-preserving logistic regression, and (3) performs information transfer among the data providers, the service provider, and the researchers. In addition, an interactive website is deployed by the service provider for researchers to conduct their studies in a secure manner and for data providers to authorize certain research requests.

#### Researchers

This includes the individuals or organizations who want to conduct research on multiple data providers’ data sets. Researchers submit their requests to the service provider, which are then sent to the data providers for further processing.

As we use threshold homomorphic encryption to guarantee data and model security, in our proposed protocol, one public key corresponds to multiple secret keys, and different secret keys are distributed to different data providers and researchers. Furthermore, we assume that there exist at least one honest party and some semihonest adversaries that are capable of reading the internal information of the colluding parties while not deviating from the defined protocol [24].

### Logistic Regression

Logistic regression is a classification algorithm that is widely used in medicine, including for disease diagnosis, clinical decision support, and risk assessment. Suppose a data set consists of pairs (*x_i_*, *y_i_*), for *i*=1,...,*N*, where *x_i_* denotes a vector of input features *x_i_*=(*x_i_^1^*,...,*x_i_^d^*) and *y_i_* is the class label. We then have:



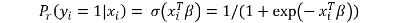



In the sigmoid function σ(*x_i_^T^β*), *β*=(*β_0_*,*β_1_*,...,*β_d_*) are the model parameters. By training a logistic regression model through minimization of the following cost function, we can obtain the optimal model parameters:



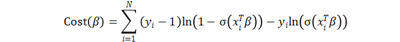



### Homomorphic Encryption

Homomorphic encryption is a special type of encryption scheme that allows computations on ciphertexts without the need to access a secret key. Once the result of the computation is decrypted, it matches the result of the operations as if they were performed on the plaintext.

In our proposed protocol, we use a ring learning with errors (RLWE)–based, somewhat homomorphic encryption scheme, called Brakerski/Fan-Vercauteren (BFV) and which supports a limited number of additions and multiplications, to perform secure multiparty logistic regression [25,26]. The BFV scheme has some helpful properties for our protocol. First, it is more practical than the other 2 types of homomorphic encryption schemes, namely, partial and fully homomorphic encryption. More specifically, fully homomorphic encryption requires time-consuming bootstrapping to support an unlimited number of operations, whereas partial homomorphic encryption allows only addition or multiplication between ciphertexts. For example, the Paillier scheme only supports addition between ciphertexts, meaning that a ciphertext can only be multiplied by a plaintext, which results in massive transfer consumption if a large number of multiplications and the security of the plaintext are required [27]. Furthermore, some optimization techniques can be used to greatly improve the computation performance in the BFV scheme as long as we set the encryption parameters properly, such as number theoretic transform (NTT) and Chinese remainder theorem (CRT) batching [28]. Finally, the BFV scheme can be extended to threshold homomorphic encryption for secure multiparty computations.

The details of the threshold variant of the BFV scheme are described as follows. The security and noise analysis of the scheme are provided in Multimedia Appendix 1 [25,29]:

1. setup(1^λ^): takes the security parameter λ as an input and returns the public parameterization param, including the degree of polynomial modulus n, the coefficient modulus q, the plaintext modulus t, and the (key, error) distribution (D1, D2).
2. THE.keygenSP(param): the service provider samples a ← R_q_ and outputs it. Here, R_q_=Z_q_[x]/(x^n^+1) is the ciphertext space of param.
3. THE.keygenSkpk(param, a): each party p_i_ samples s_i_ ← D1, e_i_ ← D2, sets si as its secret key and outputs its public key pk_i_=[−(a · s_i_+e_i_)]_q_. Let subscript *_co_ denote the combined key. The combined public key pk_co_ among parties p_1_,...,p_z_ is then computed as follows: 
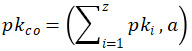
4. THE.keygenRelin(param, s_1_,...,s_z_): the parties together with the service provider generate the combined relinearization key rlk_co_. As the generation of the relinearization key is rather complicated, we will show the details of this step later.
5. THE.encrypt(m, pk_co_): This takes a polynomial m∈R_t_ as the input, where R_t_ is the plaintext space of the param. Let pk_co_=(pk_co_(0), pk_co_(1)) and Δ=⌊q/t⌋, and sample u ← D1 and (e_1_, e_2_) ← D_2_, then return:

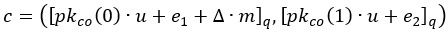

6. THE.eval(C, rlk_co_, c_1_,...,c_c_): given a circuit C, a tuple of ciphertexts encrypted by the same public key, and the corresponding relinearization key, this outputs a ciphertext c_out_. The procedure for homomorphic addition and multiplication is the same as that in the original single-key BFV scheme.
7. THE.decrypt(c, s_1_,...,s_z_): given the ciphertext c=(c(0), c(1)) encrypted by pk_co_ and the corresponding secret keys, sample (e_1_, e_z_) ← D_smg_. Here, the subscript *_smg_ means that the variance of the noise distribution is much larger than that of the input ciphertext noise distribution to guarantee circuit privacy through smudging techniques [22]. The partial decryption shares are then computed as follows:

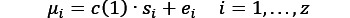

  These shares are sent to the party that requires the unencrypted result. The decryption result *m* is obtained by

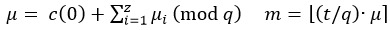

### Workflow of the Presented Protocol

**Figure 2:**
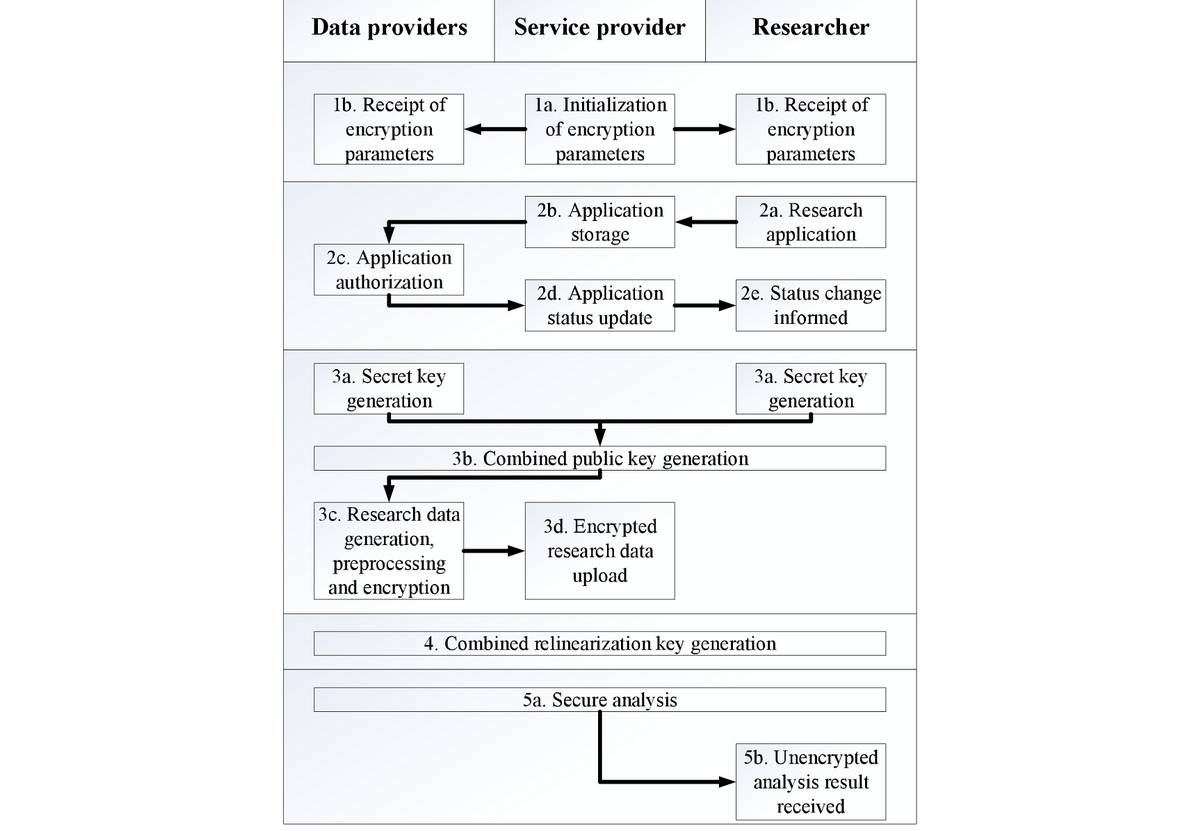
Workflow of the proposed protocol.

The workflow of our proposed protocol consists of 5 major steps, as shown in Figure 2.

#### Initialization of Encryption Parameters

The service provider initializes the BFV homomorphic encryption parameters. These parameters should be carefully selected because they affect many aspects of the encryption scheme, such as operational performance, security level, multiplicative depth of the circuit, and space consumption. Two sets of parameters must be initialized by the service provider, one for the privacy-preserving logistic regression—*param1*=(*n1*, *q1*, *t1*, *D1_1_*, *D1_2_*) and the other for the generation of the relinearization key in a secure manner—*param2*=(*n2*, *q2*, *t2*, *D2_1_*, *D2_2_*). Once initialized, the 2 sets of parameters are sent to the data providers and researchers.

To make the encryption scheme practical, these parameters should meet the following criteria. First, the degree of polynomial modulus *n* must be a power of 2. Second, the coefficient modulus and the plaintext modulus must be either a prime *P* that satisfies *P*=1 (mod 2*n*) or a composite number that is a product of distinct primes, where every prime satisfies the above condition. After setting appropriate encryption parameters, NTT can be used to accelerate the multiplications between polynomials from o(*n^2^*) to o(*n*log*n*), whereas the adoption of CRT can improve the performance of the multiplications and additions of large integers, accelerating the multiplication and addition of the polynomials [30]. More importantly, we can apply CRT batching to greatly reduce space and computational consumption. Given a certain degree of polynomial modulus *n*, we can pack up to *n* values into one polynomial using CRT batching and apply the arithmetic operations to all the values within this polynomial in a single instruction, multiple data (SIMD) manner, whereas in a naive manner, we place a single value into one polynomial and apply operations to only one value.

Furthermore, to generate relinearization keys safely and correctly, the 2 sets of parameters must satisfy the following requirements: (1) their polynomial moduli must share the same degree and (2) the plaintext modulus in *param2* must be equal to the coefficient modulus in *param1*.

#### Research Application

The research application consists of several message transfers among the data providers, service providers, and researchers. First, a researcher visits the website deployed by the service provider and sets up a new research study. When the research begins, 3 settings must be confirmed by the researcher: first, the query condition used to obtain the research data; second, the list of data providers from which the researcher wishes to obtain the research data; finally, the settings of the secure logistic regression, including the variables to be used as features and the variable to be used as a class label and the settings of the maximum number of iterations, learning rate, and termination condition of the model training. This information is stored in the database of the service provider and sent to the corresponding data providers as a research request. After receiving the request, the data providers decide whether to authorize this research and send their decision to the service provider to inform the corresponding researcher about the authorization status.

#### Key Generation and Data Preparation

Once the data providers complete the research authorization, key generation is implemented by an interactive protocol among all parties, which comprises 2 steps—THE.keygenSP and THE.keygenSkpk. After this procedure, each party *p_i_* holds its secret keys *s1_i_* and *s2_i_*, whereas 2 corresponding public keys *pk1_co_* and *pk2_co_* are broadcasted among all parties. Here, the number in the symbol represents the set of parameters to which these keys belong.

The data preparation phase then begins, which is described as follows:

1. The data provider generates their own research data according to the query condition of the research. Next, all the floating-point numbers in the research data are scaled and rounded into integers because all the operations in the BFV scheme are integer based. Categorical features are encoded as integers if they are Boolean or ordered; otherwise, one-hot encoding is implemented.
2. The data provider encodes the research data by CRT batching. As mentioned before, we can pack multiple values into one polynomial and apply operations to them in an SIMD manner via CRT batching. This means that when given a data set with d features and N samples, one can pack them into d+1 polynomials (d features and 1 class label) as long as the degrees of the polynomial moduli are larger than N.
3. The data provider encrypts all the CRT-batched polynomials using the combined public key pk1_co_. After all the plaintext polynomials are encrypted, they are sent to the service provider.

#### Relinearization Key Generation

After data preparation, the researcher, and all involved data providers together with the service provider generate the combined relinearization key. The relinearization step is not necessary for the correctness of homomorphic multiplication but is essential in our threshold-variant BFV scheme. By performing relinearization after every homomorphic multiplication, the size of the ciphertext can be strictly kept at 2, which simplifies decryption.

The relinearization key generation procedure is illustrated next. We denote the number of parties by *z*. Suppose the coefficient modulus in *param1* is a product of *k* distinct primes, whereas each party *p_i_* holds 2 secret keys *s1_i_* and *s2_i_* from *param1* and *param2*, respectively. Given a combined public key *pk2_co_* from *param2*, the following is observed:

1. Each party p_i_ performs THE.encrypt(s1_i_, pk2_co_) and outputs k ciphertexts, of which the plaintext modulus is a group of primes whose product is the coefficient modulus in param1. The ciphertexts of secret key c_j_(s1_i_) (j=1,...,k) are then sent to the service provider.
2. The service provider computes the ciphertexts of the combined secret key c_j_(s1_co_) (j=1,...,k) and sends them to the data provider and researcher:

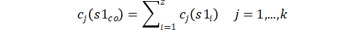

3. Each party p_i_ computes the ciphertexts of the product of the combined secret key and its secret key from *param1* as follows and sends the result to the service provider:

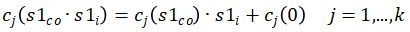

  Here, *c_j_*(0) (*j*=1,...,*k*) are the ciphertexts of 0, which contain sufficiently large noise to guarantee function privacy [31].
4. The service provider computes the ciphertexts of the square of the combined secret key c_j_(s1_co^2^_) (j=1,...,k) as follows:

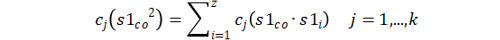

  Having encrypted the combined secret key and its square, the service provider defines the decomposition bit count *T* and the size of the relinearization key *L*=⌊log_2_(*q1*)/*T*⌋, samples *a_0_* ~ *a_L_* ← *R_q1_*, whereas each party *p_i_* samples *e_i0_* ~ *e_iL_* ← *D1_2_*, performs THE.encrypt(*e_i0_* ~ *e_iL_*, *pk2_co_*) and sends these ciphertexts *c_j_*(*e_i0_* ~ *e_iL_*) (*j*=1,...,*k*) to the service provider. After receiving encrypted noise, the service provider computes the following:

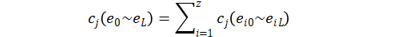

  The encrypted combined relinearization key is then generated as follows: all parties perform THE.decrypt(*c_j_*(*rlk_co_*), *s2_1_*,...,*s2_z_*) and finally return the plaintext combined relinearization key *rlk_co_*. Compared with the combined relinearization key generation procedure presented in the study by Mouchet et al [22], our method involves more transfer consumption but much less noise, which grows only linearly with the number of parties

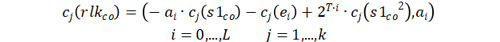

#### Privacy-Preserving Model Training and Evaluation

Secure logistic regression model training begins once all the encrypted research data and the combined relinearization key are sent to the service provider. We choose the gradient descent algorithm to train the model with homomorphically encrypted data because we can implement the algorithm using only addition and multiplication, which all fully and somewhat homomorphic encryption schemes naturally have, whereas despite its faster convergence, Newton method requires matrix inversion, which may have a very high time cost under the homomorphic encryption computation [32].

After choosing the proper training method, another major problem is the evaluation of the sigmoid function σ(*x^T^β*), because the BFV scheme can only be used to evaluate polynomial functions. Instead of simply using the Taylor polynomial to approximate the sigmoid function, we use the degree-3 least squares approximation of the sigmoid function over the interval (−8, 8), as the former has a much larger error as |*x^T^β*| increases, whereas the latter only has a small error as long as *x^T^β* is within the interval [13]. The least squares approximation polynomial is:



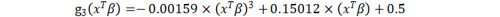



As the BFV scheme is based on integers, we apply scaling factor (SF) to scale up the floating-point number *x^T^β* into the integer ⌊*x^T^β*×*SF*⌉. In our privacy-preserving logistic regression protocol, we set *SF*=1000, which is a trade-off between approximation accuracy and performance. Specifically, if we set *SF* smaller, the approximation accuracy will decrease; if we set *SF* larger, 2 or more polynomials may be required to represent a set of values, or larger encryption parameters may be required to maintain the same multiplicative depth for a given security level, both of which result in larger space and computational resource consumption. This SF also scales up the approximation interval from (−8, 8) to (−8000, 8000), scaling the degree-1 and degree-3 coefficients to 1/1000 and 1/1000^3^, respectively, of the original value. Finally, the least squares approximation function is integerized to be compatible with the homomorphic encryption computation:



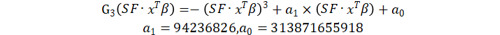



The integerized function output is then transformed into an original function:



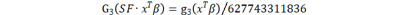



We now describe the detailed process of secure logistic regression. Before training begins, the involved data providers divide their own research data into 10 folds from *Fold1*~*Fold10* for 10-fold cross-validation and then encode the information into a vector. For example, a data set *x* containing 20 samples is divided as follows:

*Fold1* ~ (*x_1_*, *x_6_*), *Fold2* ~ (*x_2_*, *x_17_*), *Fold3* ~ (*x_3_*, *x_13_*), *Fold4* ~ (*x_4_*, *x_10_*), *Fold5* ~ (*x_5_*, *x_20_*), *Fold6* ~ (*x_7_*, *x_16_*), *Fold7* ~ (*x_8_*, *x_14_*), *Fold8* ~ (*x_9_*, *x_11_*), *Fold9* ~ (*x_12_*, *x_18_*), *Fold10* ~ (*x_15_*, *x_19_*)

Next, the information is encoded into a vector of values (1, 2, 3, 4, 5, 1, 6, 7, 8, 4, 8, 9, 3, 7, 10, 6, 2, 9, 10, 5). The vector can be viewed as a special column of research data, although this column is not used in the computation of the approximation sigmoid function.

When all the data providers finish dividing their research data, they send these vectors to the service provider. As these vectors do not contain any sensitive information, they do not need to be further encoded into CRT-batched polynomials and encrypted.

After all preparations are completed, the model training begins, as shown in Textboxes 1-3. In Textbox 1, we use minibatch gradient descent instead of batch gradient descent because the former converges faster, and we can make full use of CRT batching by simultaneously training 10 models for 10-fold cross-validation, which vastly reduces the time cost of model training. Specifically, for each iteration, the researcher assigns the sets of parameters to the research samples according to the number of iterations and the fold to which these samples belong, which means that in one iteration, a one-to-one correspondence exists between the 10 sets of parameters and the 10 folds of research data. Once the gradient ciphertexts are computed, all data providers will mask them via randomly generated encrypted noises (Textbox 3). The masked gradients are then decrypted, and only the researcher can obtain the plaintext result. As the researcher only knows the sum of noises for each fold, the correct overall gradients are finally obtained to update the model parameters of the researcher without revealing the gradient of any single sample.

**Privacy-preserving logistic regression model training.**

Input: *epoch* (# of iterations), *α* (learning rate), *ε* (step tolerance), *c*(*x*)={*c*(*x^1^*),...,*c*(*x^d^*),*c*(*y*)} (encrypted research data), *x^d+1^* (vector describing how data providers divide their research data), *b* (# of samples in one fold), *z* (# of parties), *s1_1_* ~ *s1_z_* (secret keys), *pk1_co_* (combined public key), *β*(1) ~ *β*(10) (model parameters initialized by researcher where each *β*(*i*)=(*β*(*i*)*^0^*, *β*(*i*)*^1^*,...,*β*(*i*)*^d^*))

Output: *β_new_*(1) ~ *β_new_*(10) (trained model parameters)

Researcher does:

1: For *iter*=1 to *epoch* / 9

2: *β_old_*(1) ~ *β_old_*(10) ← *β*(1) ~ *β*(10)

3: For *cv*=1 to 9

4: *B* ← empty vector

5: For-each element *i* in *x^d+1^*

6: *B*.push_back(*β*((*i*+*cv*−1) mod 10+1))

7: End for-each

8: *B’* ← CRT-batchingEncode(*B*) // *B’*={*B’^0^*,…,*B’^d^*}

9: *c*(*B^0^*) ~ *c*(*B^d^*) ← THE.encrypt(*B’*, *pk1_co_*)

10: Wait for encrypted gradient calculation *c*(*gra^0^*) ~ *c*(*gra^d^*) // See (Textbox 2) for details

11: Wait for securely decryption of encrypted gradients *gra*(1) ~ *gra*(10) // See (Textbox 3) for details

12: *β*(1) ~ *β*(10) −= (*gra*(1) ~ *gra*(10))×*α* ÷ *b*

13: End for

14: *β_new_*(1) ~ *β_new_*(10) ← *β*(1) ~ *β*(10)

15: If (||*β_new_*−*β_old_*|| ÷ ||*β_new_*||<*ε*) then

16: return *β_new_*(1) ~ *β_new_*(10)

17: End if

18:End for

**Encrypted gradient calculation.**

Input: *c*(*B^0^*) ~ *c*(*B^d^*), *c*(*x*) // See details in (Textbox 1)

Output: *c*(*gra^0^*) ~ *c*(*gra^d^*) (encrypted gradients)

Service provider does:

1: *c*(*x^T^β*) ← *c*(*B^0^*)+*c*(*B^1^*)×*c*(*x^1^*)+...+*c*(*B^d^*)×*c*(*x^d^*)

2: *c*(*G*) ← G_3_(*c*(*x^T^β*)) // G_3_ is an integerized sigmoid function

3: *c*(*gra^0^*) ~ *c*(*gra^d^*) ← [*c*(*G*)−627743311836×*c*(*y*)]×[*c*(*x^0^*) ~ *c*(*x^d^*)] // Here, *c*(*x^0^*)=1

**Secure decryption of encrypted gradients.**

Input: *x^d+1^*, *cv*, *c*(*gra^0^*) ~ *c*(*gra^d^*), *s1_1_* ~ *s1_z_*, *pk1_co_* // See details in (Textbox 1)

Output: *gra*(1) ~ *gra*(10) (unencrypted gradients)

All data providers do:

1: *e^0^* ~ *e^d^* ← random noise vectors whose size equals *x^d+1^*

2: *E*(1) ~ *E*(10) ← zero vectors whose size equals *d*+1

3: For *i*=1 to size(*x^d+1^*)

4: For *j*=1 to *d*+1

5: *E*((*x^d+1^*(*i*)+*cv*−1) mod 10+1)(*j*)+= *e^j−1^*(*i*)

6: End for

7: End for // *E*(1) ~ *E*(10) are sent to the researcher

8: *e’* ← CRT-batchingEncode(*e^0^* ~ *e^d^*)

9: *c*(*e^0^*) ~ *c*(*e^d^*) ← THE.encrypt(*e’*, *pk1_co_*) // *c*(*e^0^*) ~ *c*(*e^d^*) are sent to the service provider

Service provider does:

10:*c’*(*gra^0^*) ~ *c’*(*gra^d^*) ← *c*(*gra^0^*) ~ *c*(*gra^d^*)+*c*(*e^0^*) ~ *c*(*e^d^*)

All parties do:

11:*gra’^0^* ~ *gra’^d^* ← THE.decrypt(*c’*(*gra^0^*) ~ *c’*(*gra^d^*), *s1_1_* ~ *s1_z_*) // To ensure only the researcher obtains the plaintext result, data providers’ and researcher’s partial decryption shares are added at the service provider and the researcher, respectively.

Researcher does:

12:*gra*(1) ~ *gra*(10) ← zero vectors whose size equals *d*+1

13:*gra’’^0^* ~ *gra’’^d^* ← CRT-batchingDecode(*gra’^0^* ~ *gra’^d^*) // Decoding result is vectors whose size equals *x^d+1^*.

14:For *i*=1 to size(*x^d+1^*)

15: For *j*=1 to *d*+1

16: *gra*((*x^d+1^*(*i*)+*cv*−1) mod 10+1)(*j*)+= *gra’’^j^*^−^*^1^*(*i*)

17: End for

18:End for

19:*gra*(1) ~ *gra*(10) −= *E*(1) ~ *E*(10)

Once the model training is completed, all involved data providers encode their own research data for each fold into CRT-batched polynomials whose slots are randomly chosen to contain samples. In the meantime, the data providers also generate vectors containing information about whether a certain slot contains a sample and encode them into CRT-batched polynomials. For instance, for a CRT-batched polynomial containing samples in slots (1, 6, 8), the vector should be (1, 0, 0, 0, 0, 1, 0, 1). These polynomials are then encrypted by *pk1_co_* and sent to the service provider.

When all the aforementioned preparations are completed, the model evaluation starts, as shown in Textboxes 4-6. In Textbox 5, lines 3-5, all data providers mask the encrypted predictive values. Here, the noise generation should meet 2 criteria, whereas the noise generation in Textbox 3 line 1 has no special limitations as long as the error is random and sufficiently large to mask the true values. First, in the empty slots, we sample noise from a uniform distribution whose upper and lower bounds are the minimum and maximum values of the integerized approximation sigmoid function G_3_. Second, in the slots containing samples, we sample noise from a uniform distribution (−1569358279, 1569358279) whose corresponding values are (−0.005, 0.005) in the scaled down plaintext. In Textbox 6, lines 1-3, all data providers perform another masking; this time, the noise generation is exactly the same as in Textbox 3 line 1.

**Model evaluation.**

Input: *c*(1)(*x*, *y*) ~ *c*(10)(*x*, *y*) (10 encrypted folds of research data), *c*(1)(*x^d+1^*) ~ *c*(10)(*x^d+1^*) (encrypted vectors indicating whether a certain slot contains a sample), *β*(1) ~ *β*(10) (trained sets of parameters), *s1_1_* ~ *s1_z_* (secret keys), *pk1_co_* (combined public key)

Output: *TP*, *FP*, *TN*, *FN* (number of true positives, false positives, true negatives, and false negatives, respectively, under different predictive value thresholds)

Researcher does:

1: For *FD*=1 to 10

2: *c*(*β^0^*) ~ *c*(*β^d^*) ← THE.encrypt(*β*(*FD*), *pk1_co_*) // *c*(*β^0^*) ~ *c*(*β^d^*) are sent to the service provider

3: Wait for masked predictive values *σ* // See (Textbox 5) for details

4: For *V*=min(*G_3_*) : (max(*G_3_*)–min(*G_3_*))/100 : max(*G_3_*)

5: *X* ← empty vector

6: For-each predictive value *σ_i_* in *σ*

7: *X*.push_back(if(*σ_i_*≥*V*))

8: End for-each

9: *X’* ← CRT-batchingEncode(*X*)

10: *c*(*TP*), *c*(*FP*), *c*(*TN*), *c*(*FN*) ← *c*(*FD*)(*y*)×*X’*×*c*(*FD*)(*x^d+1^*), (1−*c*(*FD*)(*y*))×*X’*×*c*(*FD*)(*x^d+1^*), (1−*c*(*FD*)(*y*))×(1−*X’*)×*c*(*FD*)(*x^d+1^*), *c*(*FD*)(*y*)×(1−*X’*)×*c*(*FD*)(*x^d+1^*) // These 4 ciphertexts are sent to the service provider

11: Wait for masked model evaluation results *TP’*, *FP’*, *TN’*, *FN’* // See (Textbox 6) for details

12: *TP’’*, *FP’’*, *TN’’*, *FN’’* ← CRT-batchingDecode(*TP’*, *FP’*, *TN’*, *FN’*)

13: *TP*, *FP*, *TN*, *FN* ← *TP’’*−sum(*e_TP_*), *FP’’*−sum(*e_FP_*), *TN’’*−sum(*e_TN_*), *FN’’*−sum(*e_FN_*)

14: output *TP*, *FP*, *TN*, *FN* // under fold *FD* and predictive value threshold *V*

15: End for

16:End for

**Calculation of masked predictive values.**

Input: *c*(1)(*x*) ~ *c*(10)(*x*), *x^d+1^*(1) ~ *x^d+1^*(10), *c*(*β^0^*) ~ *c*(*β^d^*), *FD*, *pk1_co_*, *s1_1_* ~ *s1_z_* // See details in (Textbox 4)

Output: *σ* (masked predictive values)

Service provider does:

1: *c*(*x^T^β*) ← *c*(*β^0^*)+*c*(*β^1^*)×*c*(*FD*)(*x^1^*)+...+*c*(*β^d^*)×*c*(*FD*)(*x^d^*)

2: *c*(*G*) ← G_3_(*c*(*x^T^β*)) // G_3_ is an integerized sigmoid function

All data providers do:

3: *e* ← random noise vectors whose size equals *x^d+1^*(*FD*)

4: *e’* ← CRT-batchingEncode(*e*)

5: *c*(*e’*) ← THE.encrypt(*e’*, *pk1_co_*) // *c*(*e’*) are sent to the service provider

Service provider does:

6: *c’*(*G*) ← *c*(*G*)+*c*(*e’*)

All parties do:

7: *σ* ← CRT-batchingDecode(THE.decrypt(*c’*(*G*), *s1_1_* ~ *s1_z_*)) // The same as in (Textbox 3), only the researcher obtains the plaintext result

**Calculation of masked model evaluation results.**

Input: *c*(1)(*x^d+1^*) ~ *c*(10)(*x^d+1^*), *FD*, *pk1_co_*, *c*(*TP*), *c*(*FP*), *c*(*TN*), *c*(*FN*), *s1_1_* ~ *s1_z_* // See details in (Textbox 4)

Output: *TP’*, *FP’*, *TN’*, *FN’* (masked model evaluation results)

All data providers do:

1: *e_TP_*, *e_FP_*, *e_TN_*, *e_FN_* ← random noise vectors whose size equals to *c*(*FD*)(*x^d+1^*) // The sums of noises sum(*e_TP_*), sum(*e_FP_*), sum(*e_TN_*), sum(*e_FN_*) are sent to the researcher

2: *e’_TP_*, *e’_FP_*, *e’_TN_*, *e’_FN_* ← CRT-batchingEncode(*e_TP_*, *e_FP_*, *e_TN_*, *e_FN_*)

3: *c*(*e’_TP_*), *c*(*e’_FP_*), *c*(*e’_TN_*), *c*(*e’_FN_*) ← THE.encrypt((*e’_TP_*, *e’_FP_*, *e’_TN_*, *e’_FN_*), *pk1_co_*) // These encrypted noises are sent to the service provider

Service provider does:

4: *c’*(*TP*), *c’*(*FP*), *c’*(*TN*), *c’*(*FN*) ← *c*(*TP*)+*c*(*e_TP_*), *c*(*FP*)+*c*(*e_FP_*), *c*(*TN*)+*c*(*e_TN_*), *c*(*FN*)+*c*(*e_FN_*)

All parties do:

5: *TP’*, *FP’*, *TN’*, *FN’* ← THE.decrypt((*c’*(*TP*), *c’*(*FP*), *c’*(*TN*), *c’*(*FN*)), *s1_1_* ~ *s1_z_*) // The same as in (Textbox 3), only the researcher obtains the plaintext result

Once the model evaluation ends, the researcher obtains the number of true positives (TPs), false positives (FPs), true negatives (TNs), and false negatives (FNs) for the 10 folds and different predictive value thresholds, which should be sufficient to evaluate the trained model via 10-fold cross-validation.

## Results

### Overview

In this section, we consider the following aspects to assess the performance of our proposed multicenter secure logistic regression protocol: (1) Security analysis: security of sensitive research data and learned model; (2) accuracy loss: the loss in accuracy during the model training and evaluation with respect to the nonsecure method with real medical data; (3) model training and evaluation time: the time needed to perform 10-fold cross-validation with real medical data; and (4) scalability: how the model training and evaluation time increases as the size of the data increases in the synthetic data set.

The biomedical data sets used for the experiments are shown in Table 1 [33,34]. For the breast cancer data set, we eliminate missing samples, use all the attributes except breast-quad, and assume that the data set is provided by 1 data provider. For the surveillance, epidemiology, and end results colorectal cancer data set, we choose a portion of the samples and use 5-year survival status as the label. Moreover, all the attributes, except the registry, are used, and we assume that the data set is provided by 3 different data providers. More details about these 2 data sets are provided in Multimedia Appendix 1. We use 10-fold cross-validation, which partitions the data sets into 10 folds of approximately equal size by stratified sampling to ensure that the positive/negative ratio of each fold is approximately equal. Each time, 9 folds are used as the training set and the remaining fold is used as the test set. In addition, we assume that during model training, all data ciphertexts share the same data division vector so that the gradient ciphertexts can be summed to reduce the size of transferred data in Textbox 3 line 11.

**Table 1:** Description of the data sets.

Data sets	SEER^a^ CRC^b^ data [31]	UCI^c^ breast cancer [32]
Samples, n	49152	277
Attributes, n	10	9
Size of ciphertexts, MB	60.0	18.0

^a^SEER: surveillance, epidemiology, and end results.

^b^CRC: colorectal cancer.

^c^UCI: unique client identifier.

To set the homomorphic encryption parameters, we select the following parameters to guarantee sufficient security, as shown in Table 2. Our values for the polynomial modulus, coefficient modulus, and security level match the most recent homomorphic encryption security standards proposed by the Homomorphic-Encryption.org group [35]. The degree of polynomial modulus *n* is a power of 2, whereas the coefficient moduli in *param1* and *param2* are products of 8 and 5 distinct primes, respectively, where every prime *P* is at most 60 bits long and satisfies *P*=1 (mod 2*n*), which makes the NTT accessible. The plaintext modulus in *param1* also satisfies *t1*=1 (mod 2*n*), allowing for the implementation of CRT batching.

**Table 2:** Select parameters for Brakerski/Fan-Vercauteren homomorphic encryption.

Parameters	*param1*	*param2*
Polynomial modulus	16,384	16,384
Coefficient modulus	438-bit integer	300-bit integer
Plaintext modulus	1125899904679937	Coefficient modulus of *param1*
Key distribution	Uniform distribution {−1, 0, 1}	Uniform distribution {−1, 0, 1}
Error distribution	Discrete Gaussian distribution, with σ=3.2	Discrete Gaussian distribution with σ=3.2
Security level	128-bit	192-bit

To simulate a real-world scenario, we place the data providers, the researcher, and the service provider on different machines. For the data providers and the researcher, we use PCs with a 2.2-GHz Intel Core i7-8750H processor and 16.0 GB RAM (Windows 10 Enterprise). For the service provider, we use a server with a 2.3 GHz Intel Xeon Gold 6140 processor and 128.0 GB RAM (Linux 3.10.0). The secure logistic regression protocol is implemented in C++ using Microsoft SEAL v3.0 and is publicly available at GitHub [36], where we made some modifications to support the threshold-variant BFV scheme [37]. All PCs have an internet connection of 100 Mbps bandwidth.

### Security Analysis

In our protocol, security means that corrupted parties will not be able to obtain sensitive data or learned models from honest parties. Here, we show the security of our protocol from the following 2 aspects: (1) honest parties’ secret keys will not be obtained by the corrupted parties so that no ciphertext will be decrypted illegally, including the encrypted data, model parameters, and any other intermediate results and (2) if the researcher is an adversary, he or she cannot obtain any meaningful information about honest parties’ individuals from the unencrypted intermediate results.

#### Security of Secret Keys

To demonstrate the security of the secret keys, we use the simulation paradigm described in the study by Goldreich [38], that is, for all adversaries, there exists a simulator program *S* that, when provided only with the adversaries’ input and output, can simulate the adversaries’ view in the protocol, and the simulated view is computationally indistinguishable from the real view. Suppose there are *z* parties. Let *A* denote the adversaries, defined as a subset of at most *z* −1 corrupted parties, and *H* denote the honest parties.

#### Combined Public Key Generation

In the generation of the combined public key, *S* can simulate the adversaries’ view of public key shares (*pk_1_*, *pk_2_*,..., *pk_z_*) by randomizing these shares under 2 constraints: (1) the simulated shares must sum to *pk_co_*(0) and (2) the adversary shares must be equal to the real shares. *S* can compute this sharing as follows:



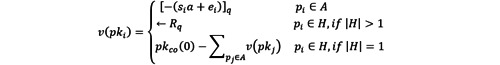



When |*H*|>1, there is no efficient algorithm that can distinguish between the simulated and real shares in *H* because of the decision-RLWE problem. When |*H*|=1, *S* computes the real shares of the honest party. However, because both *s_i_* and *e_i_* are private inputs from party *p_i_*, the adversaries cannot find the secret key of the honest party because of the search-RLWE problem.

#### Decryption

Given the ciphertext *c*=(*c*(0), *c*(1)), during the decryption process, *S* can simulate the adversaries’ view of the decryption shares (*μ_1_*, *μ_2_*,..., *μ_z_*) by randomizing these shares under 2 constraints: (1) the simulated shares must sum to *μ*–*c*(0) and (2) the adversary shares must be equal to the real shares:



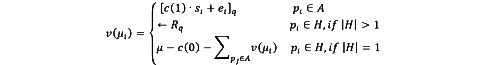



When considering the distribution of the simulated and real views alone, the RLWE assumption is sufficient to ensure the security of secret keys of *H* if the researcher is uncorrupted. However, if the researcher becomes an adversary, they can extract the noise of *c* as follows:



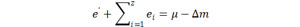



where *e’* is the noise of *c*, which should be unknown to the researcher; otherwise, the RLWE assumption will be broken and the secret keys of the honest parties may be exposed to the researcher. Let *var^2^_c_* denote the variance of a centered Gaussian distribution that *e* follows and *var^2^_smg_* denote the variance of *D_smg_*, which is used to generate *e_i_*. Thus, as long as the ratio *var^2^_c_*/*var^2^_smg_* is negligible, the following 2 distributions are statistically indistinguishable, which means that *e’* is unknown to the researcher and that the researcher cannot obtain *H*’s secret keys:



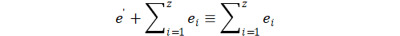



#### Unencrypted Intermediate Results

First, during model training, all data providers apply one-time-use noise to mask the encrypted gradient before decryption, meaning that even if only one data owner is honest, it will not lead to the disclosure of the gradients of the individuals.

Second, during model evaluation, the researcher will inevitably obtain CRT-batched polynomials containing the predictive values for each sample. Given a masked predictive value *σ_i_* ∈ (*V_j_*, *V_j+1_*), the probability of recovering the research data is computed as follows:



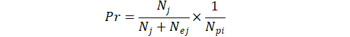



Here, *N_j_* is the number of samples whose predictive value belongs to (*V_j_*, *V_j+1_*), *N_ej_* is the number of empty slots whose value belongs to (*V_j_*, *V_j+1_*), and *N_pi_* is the number of all possible combinations of feature values whose predictive value belongs to (*σ_i_*−1569358279, *σ_i_*+1569358279). Therefore, as long as either of these 2 terms is sufficiently small, it is impossible for the researcher to recover the feature values.

Furthermore, because the encrypted (TP, FP, TN, and FN) information of samples under different predictive value thresholds is also masked by all data providers before being sent to the researcher, the researcher cannot obtain the label of any specific sample.

### Accuracy Loss

**Figure 3:**
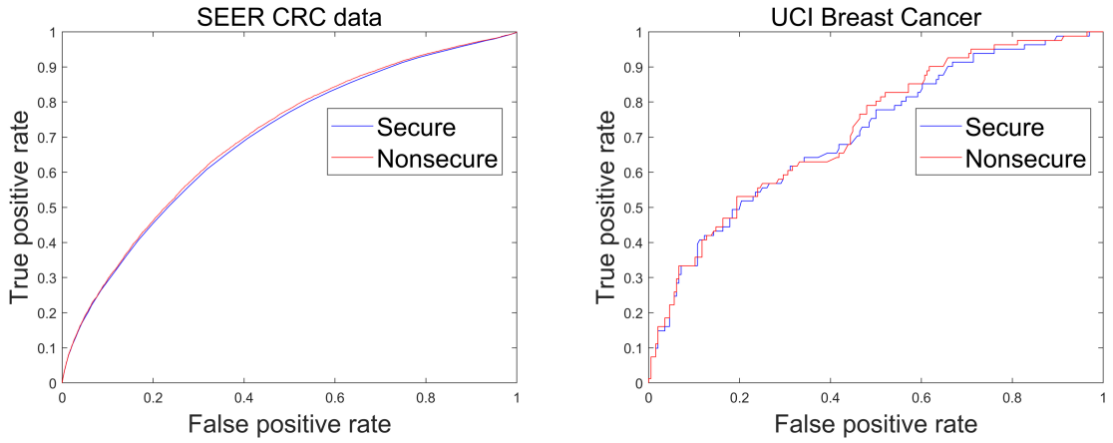
Average receiver operating characteristic curves of nonsecure and proposed secure logistic regressions. CRC: colorectal cancer; ROC: receiver operating characteristic; SEER: surveillance, epidemiology, and end results; UCI: University of California, Irvine.

In Table 3, we demonstrate the accuracy of our protocol by comparing the area under the curve between the nonsecure logistic regression and our secure logistic regression, where the former uses the standard sigmoid function and both have the same hyperparameters (learning rate *α*=.1, 45 iterations). Compared with that of the nonsecure protocol, a relatively small loss of accuracy was observed in our protocol, which was not statistically significant (the smallest *P*=.09). The average receiver operating characteristic curves from the 10-fold cross-validation are plotted in Figure 3.

**Table 3:** Accuracy comparison between nonsecure and proposed secure logistic regressions.

Data sets	SEER^a^ CRC^b^ data	Breast cancer
AUC^c^ (nonsecure)	0.703 (0.008)	0.728 (0.156)
AUC (our protocol)	0.696 (0.008)	0.717 (0.164)
*P* value (AUC)	.09	.88
Accuracy (nonsecure)	0.620 (0.013)	0.664 (0.149)
Accuracy (our protocol)	0.612 (0.013)	0.632 (0.155)
*P* value (accuracy)	.18	.64
*F*_1_^d^ (nonsecure)	0.654 (0.012)	0.508 (0.198)
*F*_1_ (our protocol)	0.649 (0.012)	0.505 (0.240)
*P* value (*F*_1_)	.42	.97

^a^SEER: surveillance, epidemiology, and end results.

^b^CRC: colorectal cancer.

^c^AUC: area under the curve.

^d^*F*_1_: the harmonic mean of the precision and recall.

Furthermore, in Table 4, we test the relationships between the learning rate and the convergence of the nonsecure and secure logistic regressions. Although our protocol’s model training will be fully spoiled because of the limited valid input interval for the approximation sigmoid function when the learning rate becomes too large, our protocol has a slightly broader range of learning rate selection than the nonsecure protocol.

**Table 4:** ||βnew–βold|| ÷ ||βnew|| after 99 iterations (surveillance, epidemiology, and end results colorectal cancer data).

Learning rate	0.1	0.2	0.3	0.4
Nonsecure	0.056	0.046	0.302	0.347
Our protocol	0.061	0.052	0.047	—^a^

^a^Fail to convergence.

### Model Training and Evaluation Time

We show the time consumption of the 10-fold cross-validation for the 2 different data sets in Table 5.

Here, we compare our protocol with the SecureLR protocol by Jiang et al [19], which is also optimized with NTT and CRT batching but evaluated on only 1 PC. As shown in their experiments, SecureLR can train only 1 model at a time and requires 44.9 seconds per iteration over a data set with a ciphertext size of 5.0 M. In comparison, our protocol can train 10 models simultaneously and perform each iteration much faster (on a data set with a ciphertext size of 60.0 M in less than 10 seconds per iteration). Moreover, our protocol supports secure model evaluation with reasonable time consumption.

**Table 5:** Time consumption of the proposed protocol.

Data sets	Iterations, n	Training time	Time per iteration (seconds)	Evaluation time
SEER^a^ CRC^b^ data	45	7 min 29 seconds	9.98	20 min 27 seconds
UCI^c^ breast cancer	45	4 min 24 seconds	5.87	14 min 28 seconds

^a^SEER: surveillance, epidemiology, and end results.

^b^CRC: colorectal cancer.

^c^UCI: unique client identifier.

### Scalability Evaluation

To test our protocol’s scalability, we use a synthetic data set with different numbers of data providers and features, as shown in Tables 6 and 7. Given a certain number of features *d*, for the sake of simplicity, we suppose that every data provider encrypts (*d*+1) polynomials. As the number of data providers increases, the computation times of both the model training and evaluation increase proportionally, whereas there is no increase in the transfer time of the model training because the size of the transferred data (encrypted parameters and gradients) is only related to the number of features. Similarly, because there is no relationship between the number of data providers and the transfer of the encrypted (TP, FP, TN, and FN), the transfer time of the model evaluation increases very less. As the number of features increases, the computation and transfer times of the model training increase proportionally, whereas the computation and transfer times of the model evaluation increase only slightly because the majority of the model evaluation involves the computation of (TP, FP, TN, and FN) information under different predictive value thresholds, which is not related to the number of features.

**Table 6:** Scalability of the proposed protocol for different numbers of data providers (9 features).

Data providers, n	Size of ciphertexts, MB	Iterations, n	Training time (computation)	Training time (transfer)	Evaluation time (computation)	Evaluation time (transfer)
3	60.0	45	4 min 16 seconds	3 min 13 seconds	9 min 54 seconds	10 min 33 seconds
5	100.0	45	6 min 26 seconds	3 min 13 seconds	15 min 24 seconds	10 min 39 seconds
10	200.0	45	12 min 45 seconds	3 min 12 seconds	30 min 42 seconds	10 min 51 seconds
15	300.0	45	19 min 5 seconds	3 min 13 seconds	45 min 54 seconds	11 min 3 seconds
20	400.0	45	25 min 52 seconds	3 min 13 seconds	61 min 13 seconds	11 min 17 seconds

**Table 7:** Scalability of the proposed protocol for different numbers of features (3 data providers).

Features, n	Size of ciphertexts, MB	Iterations, n	Training time (computation)	Training time (transfer)	Evaluation time (computation)	Evaluation time (transfer)
3	60.0	45	4 min 16 seconds	3 min 13 seconds	9 min 54 seconds	10 min 33 seconds
5	100.0	45	8 min 30 seconds	6 min 23 seconds	10 min 22 seconds	10 min 53 seconds
10	200.0	45	12 min 48 seconds	9 min 37 seconds	10 min 47 seconds	11 min 13 seconds
15	300.0	45	16 min 54 seconds	12 min 50 seconds	11 min 16 seconds	11 min 32 seconds
20	400.0	45	21 min 13 seconds	16 min 10 seconds	11 min 40 seconds	11 min 53 seconds

## Discussion

### Principal Findings

As researchers cannot obtain unencrypted research data, they may have difficulty choosing the proper hyperparameters, especially the learning rate. Despite a slightly broader range of learning rate selection, the setting of the learning rate is still very important in our privacy-preserving multicenter logistic regression protocol because compared with the nonsecure protocol, our protocol still has a considerable time cost. In our proposed protocol, interactions exist among the service provider, the data providers, and the researcher, allowing the researcher to obtain the plaintext model parameters in every iteration. As a result, the researcher can easily judge whether the hyperparameters are set properly according to the trend of the model parameters. Moreover, the researcher can halt the model training in the early stages, which results in less waste of computational resources. However, to implement the web-based protocol, clients must be installed on all the data providers’ and researchers’ machines, which must be kept online during the entire process of model training and model evaluation, leading to an additional consumption of network bandwidth.

There is a trade-off between computation and transfer consumption in our protocol. Although some solutions use fully homomorphic encryption to avoid decryption during model training [14,15], our proposed protocol uses somewhat homomorphic encryption for several reasons. First, to support an unlimited number of operations, a bootstrapping process is required, which is very time consuming. More time is consumed in threshold homomorphic encryption because we must select larger encryption parameters because there is not only greater noise in the combined public and relinearization keys but also greater smudging noise during decryption. Second, to avoid decryption, fixed-point arithmetic operations without a rounding process are required. Bonte and Vercauteren [14] use nonintegral base nonadjacent form with window size *ω* to encode a real number as a polynomial, which may affect the use of CRT batching (the most important optimization technique in our protocol), whereas Chen et al [15] use the Cheon-Kim-Kim-Song (CKKS) [39] scheme, which is also based on RLWE and naturally supports floating-point approximate arithmetic operations. However, in the CKKS scheme, the decryption result contains noise, meaning that in the threshold variant of the CKKS scheme, we must set a very high value for the encryption parameter *scale* to avoid destruction of the plaintext by the smudging noise, which greatly reduces the multiplicative depth of the circuit.

### Limitations

Our proposed protocol has a few limitations. First, to make the privacy-preserving logistic regression realistic, this protocol requires a high-speed and stable network. Second, as the BFV scheme is based on integers, before encryption, all floating-point numbers must be scaled up and rounded to integers. A larger SF can support a higher level of precision but will also result in higher computation and storage costs for a given security level. Third, in a real-world scenario, a single patient may have multiple medical records across different data providers, which rarely occurs when data providers are far apart but is not uncommon when data providers are located in the same region (eg, a city). Therefore, in the latter case, further research on privacy-preserving identification and deduplication is required to ensure that there are no duplicate medical records to affect the analysis results. Furthermore, this study mainly focuses on technical issues and thus does not delve into matters related to ethics and law, which are also very important in multiparty medical research.

### Conclusions

In this paper, we propose the first privacy-preserving multiparty logistic regression model training and evaluation protocol based on threshold homomorphic encryption. We conduct experiments in simulated real-life scenarios, and the results demonstrate that the proposed protocol is practical for real-world use. We believe that our work can help medical institutions eliminate privacy leakage concerns during data sharing, promote multicenter medical research, and thus improve the use of medical data to some extent.

In the future, we will extend our tools to be more practical. As the BFV homomorphic encryption scheme does not have indistinguishability under chosen ciphertext attack security, additional security technology, such as hashing, should be integrated into the tools to prevent malicious attackers from tampering with the ciphertexts. More privacy-preserving statistics and machine learning methods will be added to our tools to facilitate considerably enhance flexibility in secure multicenter research. Furthermore, we will improve the efficiency of our tools using graphics processing unit or field programmable gate array acceleration.

## Appendix

Multimedia Appendix 1 Details of used biomedical data, details of Brakerski/Fan-Vercauteren (BFV) threshold homomorphic encryption, security analysis, and noise analysis.

## Abbreviations

BFV: Brakerski/Fan-Vercauteren
CKKS: Cheon-Kim-Kim-Song
CRT: Chinese remainder theorem
FN: false negative
FP: false positive
NTT: number theoretic transform
RLWE: ring learning with errors
SF: scaling factor
SGX: software guard extensions
SIMD: single instruction, multiple data
TN: true negative
TP: true positive
