# Supplementary material for: Web-Based Privacy-Preserving Multicenter Medical Data Analysis Tools Via Threshold Homomorphic Encryption: Design and Development Study
Source: J Med Internet Res. 2020 Dec 8;22(12):e22555. doi: 10.2196/22555 (PMC7755539; doi:10.2196/22555)
Supplement: Multimedia Appendix 1 [file jmir_v22i12e22555_app1.docx]

Supplementary Materials: Private and Eﬃcient

Query Processing on Outsourced Genomic

Databases

Supplementary Materials: Private and Eﬃcient

Query Processing on Outsourced Genomic

Databases

Supplementary Materials

1 Details of used biomedical data

Table 1. Summary of predictors and outcome of interest used in the SEER CEC dataset.

| Characteristics | Levels —— ratio |
| --- | --- |
| Predictors | |
| Age | < 55 —— 18.78%  55 ~ 64 —— 20.84%  65 ~ 74 —— 24.05%  ≥ 75 —— 36.33% |
| Gender | Male —— 49.25%  Female —— 50.75% |
| Grade | Well differentiated —— 8.78%  Moderately differentiated —— 70.48%  Poorly differentiated OR Undifferentiated —— 20.74% |
| Histology | Adenocarcinoma —— 75.09%  Mucinous OR Signet ring cell —— 9.25%  Others —— 15.66% |
| Site | Colon —— 77.68%  Rectum —— 22.32% |
| T stage | T1 —— 8.93%  T2 —— 17.30%  T3 —— 60.42%  T4a —— 7.49%  T4b —— 5.86% |
| N stage | N0 —— 58.57%  N1a —— 12.09%  N1b/N1c —— 12.81%  N2a —— 8.73%  N2b —— 7.80% |
| Tumor size | Not a categorical variable |
| EOD10_PN | Not a categorical variable |
| Outcome | |
| 5-year survival status | Alive —— 55.13%  Dead —— 44.87% |

Table 2. Summary of predictors and outcome of interest used in the UCI BC dataset

| Characteristics | Levels —— ratio |
| --- | --- |
| Predictors | |
| Age | 20 ~ 29 —— 0.36%  30 ~ 39 —— 13.00%  40 ~ 49 —— 32.13%  50 ~ 59 —— 32.85%  60 ~ 69 —— 19.86%  70 ~ 79 —— 1.81% |
| Menopause | Lt40 —— 1.81%  Ge40 —— 44.40%  Premeno —— 53.79% |
| Tumor size | 0 ~ 4 —— 2.89%  5 ~ 9 —— 1.44%  10 ~ 14 —— 10.11%  15 ~ 19 —— 10.47%  20 ~ 24 —— 17.33%  25 ~ 29 —— 18.41%  30 ~ 34 —— 20.58%  35 ~ 39 —— 6.86%  40 ~ 44 —— 7.94%  45 ~ 49 —— 1.08%  50 ~ 54 —— 2.89% |
| Inv-nodes | 0 ~ 2 —— 75.45%  3 ~ 5 —— 12.27%  6 ~ 8 —— 6.14%  9 ~ 11 —— 2.53%  12 ~ 14 —— 1.08%  15 ~ 17 —— 2.17%  24 ~ 26 —— 0.36% |
| Node-caps | Yes —— 20.22%  No —— 79.78% |
| Deg-malig | 1 —— 23.83%  2 —— 46.57%  3 —— 29.60% |
| Breast | Left —— 52.35%  Right —— 47.65% |
| Irradiat | Yes —— 22.38%  No —— 77.62% |
| Outcome | |
| Class | No-recurrence-events —— 70.76%  Recurrence-events —— 29.24% |

2 Details of FV Threshold Homomorphic Encryption

- THE.setup(*1^λ^*): take the security parameter *λ* as an input and return the public parameterization *parm*, including the degree of polynomial modulus *n*, the coefficient modulus *q*, the plaintext modulus *t*, and the (key, error) distribution *(D_1,_ D_2_)*.
- THE.keygenSP(*parm*): the service provider samples *a ← R_q_* and outputs it. Here *R_q_ = Z_q_[x]/(x^n^ + 1)* is the ciphertext space of *parm*.
- THE.keygenSkpk(*parm*, *a*): each party *p_i_* samples *s_i_ ← D_1_*, *e_i_ ← D_2_*, sets *s_i_* as its secret key and outputs its public key *pk_i_ = [-(a · s_i_ + e_i_)]_q_*. Then the combined public key among parties *p_1_,...,p_z_* is computed as follows:

$${pk}_{co}=\left( \sum_{i=1}^{z} {pk}_{i}, a \right)$$

- THE.keygenRelin(*parm*, *s_1_,...,s_z_*): parties together with the service provider generate the combined relinearization key *rlk_co_*. Because the generation of relinearization key is rather complicated, we show the detail of this step in Section IV-D.
- THE.encrypt(*m*, *pk_co_*): take a polynomial *m ∈ R_t_* as the input where *R_t_* is the plaintext space of *parm*. Let *pk_co_ = (pk_co_[0], pk_co_[1])* and *Δ = ⌊q/t⌋*, sample *u ← D_1_* and *(e_1_, e_2_) ← D_2_*, then return

$$c=\left( \left[ {pk}_{co}\left[ 0 \right]\cdot u+e_{1}+\Delta\cdot m \right]_{q},\left[ {pk}_{co}\left[ 1 \right]\cdot u+e_{2} \right]_{q} \right)$$

- THE.eval(*C, rlk_co_, c_1_*,...,*c_c_*): given a circuit *C*, a tuple of ciphertexts encrypted by the same public key, and the corresponding relinearization key, output a ciphertext *c_out_*. The way of homomorphic addition and multiplication is the same with the original single-key FV scheme.
- THE.decrypt(*c*, *s_1_,...,s_z_*): given a ciphertext *c = (c[0], c[1])* encrypted by *pk_co_* and corresponding secret keys, sample *(e_1_,...,e_z_) ← D_smg_* whose variance is much larger than that of the input ciphertext noise distribution in order to guarantee circuit privacy by smudging techniques. Then the partial decryption shares are computed as follows:

$$\mu_{i}=c\left[ 1 \right]\cdot s_{i}+e_{i} i=1,...,z$$

These shares are sent to the party who requires the unencrypted result. It gets the decryption result *m* by:

$\mu= c\left[ 0 \right]+\sum_{i=1}^{z} \mu_{i}\left( mod q \right)$ $m=\left\lfloor\left( t/q \right) \right.\left. \cdot\mu\right\rceil$

3 Security Analysis

We analyze the security of the FV threshold homomorphic encryption in the passive adversary model. Suppose there are *z* parties. Let *P* denote all involved parties, *A* denote the adversary, defined as a subset of at most *z – 1* corrupted parties in *P*. We prove the security of the scheme in the ideal/real simulation paradigm [1]. That is, for every possible *A*, we prove by construction that there exists a simulator program *S* that, when provided only with *A*’s input and output, can simulate *A*’s view in the protocol. To achieve the privacy requirement, we require that *A* must not be able to distinguish the real view (generated from the honest parties’ inputs) from the simulated one (generated with the adversary’s input only). For a given value *x*, we denote $\tilde{x}$ its simulated equivalent. Unless otherwise stated, we consider computational indistinguishability between distributions, denoted $\tilde{x}\equiv x$.

Our threat model implies that at least one honest player exists, which we denote *P_h_*. The choice for *P_h_* when multiple honest parties exist is irrelevant and does not reduce generality. It does, however, help simplify the formulation of the security argument. We denote *H* the set *P\(A∪{P_h_})* of all other honest parties. Hence, the tuple *(A, H)* can represent any partition of *P\{P_h_}*. In particular, both *A* and *H* can be empty in the following arguments.

**A. Combined public key generation**

We consider an adversary *A*, attacking THE.keygenSkpk. Along with *s_i_*, we consider *e_i_* as private inputs to the protocol for each party *P_i_*. Thus, we model the functionality of combined public key generation as *f_cpg_({s_i_, e_i_ | P_i_*∈*P}) = (p_0_, p_1_)*.

We observe that the view of each party in the execution of combined public key generation consists in the tuple *(p_0,1_, p_0,2_,..., p_0,z_)* of all the players’ shares, which corresponds to an additive sharing of *p_0_*. *S* can simulate these shares by randomizing them under two constraints: (1) the simulated shares must sum up to *p_0_* and (2) the adversary shares must be equal to the real ones (otherwise, it could distinguish from the real ones). *S* can compute this sharing as

$$\tilde{p}_{0,i}=\left\{ \begin{aligned} \left[ -\left( s_{i}p_{1}+e_{i} \right) \right]_{q} P_{i}\in A \\ a_{i}\leftarrow R_{q} P_{i}\in H \\ \left[ p_{0}-\sum_{P_{j}\in A\cup H} \tilde{p}_{0,j} \right]_{q} P_{i}=P_{h} \end{aligned} \right.$$

To show that $\left( \tilde{p}_{0,1},\tilde{p}_{0,2},\ldots,\tilde{p}_{0,z} \right)\equiv\left( p_{0,1},p_{0,2},\ldots,p_{0,z} \right)$, we observe that any probabilistic polynomial time *A* distinguishing, with non-negligible advantage, between real and simulated shares of those players in *H* would directly yield a distinguisher for the decision-RLWE problem. For the share of player *P_h_*, we consider two cases: (1) When *H ≠ ∅*, the share *p_0,h_* is a uniformly random element in *R_q_* because $\left[ \sum_{P_{j}\in H} p_{0,j} \right]_{q}$ is itself so, and the same indistinguishability argument as above applies. (2) In the presence of *z – 1* adversaries, *H = ∅* and *S* computes the real value for the honest party’s share, hence outputting the real view.

**B. Decryption**

Let *P_k_* be the party who obtains the plaintext result. Given a ciphertext *c = (c_0_, c_1_)* encrypted by *pk_co_*, in decryption process firstly all other parties compute their partial decryption shares, which are then added to *c_o_* to get *c’ = (c’_0_, c_1_)*. We can formulate this functionality implicitly, as the computation of *c’_0_* satisfying

$$c_{0}-e+c_{1}\cdot\sum_{i=1}^{z} s_{i}={c^{'}}_{0}-e^{'}+c_{1}\cdot s_{k}$$

Where *e* and *e’* are the noise terms resulting from decryptions of *c* and *c’*, respectively. Hence, we consider its explicit form as an equivalent and minimal ideal multiparty functionality *f_pd_*, such that

$$\hat{h}=\hat{f}_{pd}\left( \left\{ s_{i},{e'}_{i} | \forall P_{i}\in P\backslash\left\{ P_{k} \right\} \right\} \right)={c^{'}}_{0}-c_{0}=e^{'}-\hat{e}+c_{1}\cdot\sum_{i} s_{i}$$

Where *c_0_*, *c_1_* are considered public, and $\hat{e}=e$ is an ideal error term cancelling *e*. This is because, ideally, the output ciphertext should look fresh. As this term cannot be efficiently computed in practice, the real output differs from the ideal one. Simulation-based proofs allow this difference, as long as we prove that the ideal and real outputs are undistinguishable for the adversary (Property 1). This formalizes the need of smudging within the security argument. Then, we show that, even when having access to the real output, the adversary cannot distinguish the simulated view from the real one (Property 2). Therefore, showing both Properties (1) and (2) implies

$$\left( \tilde{h}_{1},\tilde{h}_{2},\ldots,\tilde{h}_{z},\hat{h} \right)\equiv\left( h_{1},h_{2},\ldots,h_{z},h \right)$$

i.e., that the partial decryption protocol securely computes its functionality.

1) Output indistinguishability: We want to show that

$$e^{'}-\hat{e}+c_{1}\cdot\sum_{i} s_{i}=\hat{h}\equiv h=e^{'}+c_{1}\cdot\sum_{i} s_{i}$$

Where *h* denotes the real protocol output. If the adversary is allowed to know *s_k_*, we cannot rely on computational indistinguishability of the RLWE-like structure of *h*. More specifically, such an adversary can extract the noise from the decryption of the key-switched ciphertext, as *e + e’ = c_0_ + h + s_k_c_1_ – Δm*. Hence, we require this extracted noise to be statistically indistinguishable from that of the ideal output, where it is fresh:

$$e^{'}-e\equiv e'$$

As *e* is the error of *c*, it follows a centered Gaussian distribution whose variance we denote *σ^2^_c_*. Whereas, *e’ – e* is the sum of all the noise terms protecting the partial decryption shares, which are sampled according to the *D_smg_* that has variance *σ^2^_smg_*. Thus, as long as the ratio *σ^2^_c_/σ^2^_smg_* is negligible, the two distributions are statistically indistinguishable, which implies that $\hat{h}\equiv h$.

2) View indistinguishability: The view of any party in the partial decryption protocol is an additive sharing *(h_1_, h_2_,..., h_z_)* of *h*, which *S* can simulate as

$$\tilde{h}_{i}=\left\{ \begin{aligned} \left[ s_{i}c_{1}+{e'}_{i} \right]_{q} P_{i}\in A \\ a_{i}\leftarrow R_{q} P_{i}\in H \\ \left[ h-\sum_{P_{j}\in A\cup H} \tilde{h}_{j} \right]_{q} P_{i}=P_{h} \end{aligned} \right.$$

When considering the distribution of the simulated and real views alone, the usual decision-RLWE assumption suffices: *(s_i_c_1_ + e’_i_, c_1_)* is undistinguishable from *(a ← R_q_, c_1_)* for an adversary that does not know *s_i_* and *e’_i_*. However, we need to consider this distribution jointly with that of the real output. We recall that an adversary having access to *s_k_* can extract *e + e’* from the output, and might be able to estimate *e’_i_* for *P_i_ ∉ A*. Hence, we need to make sure that the uncertainty the adversary has in estimating *e’_i_* is sufficiently large to protect each share *h_i_* in the partial decryption protocol. This is formalized in the following assumption.

**Assumption 1.** An input ciphertext *(c_0_, c_1_)* to the partial decryption protocol is such that $c_{0}+c_{1}\cdot\sum_{i} s_{i}=\Delta\cdot m+e_{c}$ where *e_c_ = e_A_ + e_h_* includes a term *e_h_* that is unknown to, and independent from, the adversary. Furthermore *e_h_* follows a distribution according to the RLWE hardness assumptions.

If Assumption 1 holds, we know that *A* can only approximate the term *e_h_* up to an error *e_c,h_*, which is enough to make *(h_h_, c_1_)* indistinguishable from *(a ← R_q_, c_1_)*, even if the adversary controls *z − 1* parties. We remark that as long as all parties provide at least one input (for which the noise is fresh) to the homomorphic function evaluation, the requirement of Assumption 1 is satisfied.

4 Noise Analysis

We now analyze the behavior of the FV threshold homomorphic encryption in terms of noise growth. We recall that our combined relinearization key only differ from the original scheme in the magnitude of the noise they contain. Hence, the analysis of [2] still applies, with a larger worst-case error norm that we express as a function of the number of parties *z*.

We represent ciphertexts as elements of *R_q_[S]*. The infinity norm of a polynomial *p* (i.e., its largest coefficient in absolute value) is denoted *||p||* (*||p|| ≤ q/2* for *p∈R_q_*). We also recall that, since the polynomial modulus in *R_q_* is a degree-*n* power of *2* cyclotomic, we have *||ab|| ≤ n||a||||b||*. We consider an instantiation of our scheme with *z* parties.

As a result of the secret-key generation procedure, where each *s_i_* is sampled from *R_3_*, we know that *||s|| ≤ z*. It must be noted that, by assuming a trusted dealer or a more complex key-generation protocol, we could lower or completely remove the factor *z*, so we keep the norm of *s* as a parameter in the following, for the sake of generality. As a result of our combined public key generation, the combined public key noise is $e_{cpk}=\sum_{i=1}^{z} e_{i}$, which implies that *||e_cpk_|| ≤ zB*, where *B* is the worst-case norm for an error term sampled from the RLWE error distribution.

**A. Fresh encryption**

Let *c = (c_0_, c_1_)* be a fresh encryption of a message *m* under *pk*_co_, such that $\frac{t}{q}\left( c_{0}+c_{1}\cdot\sum_{i=1}^{z} s_{i} \right)=m+v_{fresh}$; we have

$$\left\| v_{fresh} \right\|\leq-\frac{r_{t}\left( q \right)}{q}\left\| m \right\|+\frac{t}{q}B\left( nz+n\left\| s \right\|+1 \right)$$

Where *r_t_(q)* denotes the remainder of the division of *q* by *t*. Thus, for a key generated by the combined public key generation, the worst-case fresh ciphertext noise is linear in the number *z* of parties.

**B. Arithmetic operations**

We consider *c_1_* and *c_2_*, two encryptions such that $\frac{t}{q}\left( c_{j0}+c_{j1}\cdot\sum_{i=1}^{z} s_{i} \right)=m_{j}+v_{j}$ with *||v_j_|| < B_j_* for *j = 1, 2*.

Let *c_add_* be the homomorphic sum of *c_1_* and *c_2_*, such that $\frac{t}{q}\left( c_{add0}+c_{add1}\cdot\sum_{i=1}^{z} s_{i} \right)=m_{1}+m_{2}+v_{add}$ with

$$\left\| v_{add} \right\|\leq B_{1}+B_{2}+t\frac{r_{t}\left( q \right)}{q}\left( m_{add}-\left[ m_{add} \right]_{t} \right)$$

Where *m_add_ = m_1_ + m_2_*, and the last term is non-zero only in the case where *||m_add_|| > t*. This follows directly from the analysis in [2].

Let *c_mul_* be the homomorphic product of *c_1_* and *c_2_*, such that $\frac{t}{q}\left( c_{mul0}+c_{mul1}\cdot\sum_{i=1}^{z} s_{i} \right)=\left[ m_{1}m_{2} \right]_{t}+v_{mul}$. Then, by relying on the upper bound given by Lemma 2 in [2], we have

$$\left\| v_{mul} \right\|\leq nt\left( B_{1}+B_{2} \right)\left( n\left\| s \right\|+1 \right)+2t^{2}n^{2}\left( \left\| s \right\|+1 \right)$$

As for the homomorphic addition, the additional noise depends only on the operands’ inherent noise and the magnitude of the secret key (there is no additional noise source). Therefore, the homomorphic multiplication noise-growth before relinearization does not directly depend on the number of parties. However, it does, indirectly, when *s* is the sum of *z* elements of *R_3_*.

**C. Relinearization**

We analyze the noise resulting from a Type I relinearization [2] performed using a combined relinearization key. Thus, for a ciphertext *c = (c_0_, c_1_, c_2_)*, we can write its *2*-component equivalent as *c_relin_ = (c’_0_, c’_1_)*, where $\frac{t}{q}\left( c_{relin0}+c_{relin1}\cdot\sum_{i=1}^{z} s_{i} \right)=m+v_{fresh}+v_{relin}$ with

$$\left\| v_{relin} \right\|\leq\frac{2^{T}nt}{2q}\left( L+1 \right)zB$$

Where *T* denotes the decomposition bit count and *L=⌊log_2_(q)⌋/T* denotes the size of the relinearization key. Therefore, the noise introduced by relinearization increases by a factor that is linear in *z*; this factor stems from the noise introduced in the relinearization key. This result is consistent, as the relinearization key generated by our scheme only differ from those of the single-user scheme in the magnitude of the noise term. Analogously to the original scheme, the noise introduced by the relinearization is independent of the noise already present in the input ciphertext.

**D. Decryption**

Let *P_k_* be the party who obtains the plaintext result, *c = (c_0_, c_1_)* be an encryption of *m* under the combined public key *pk_co_*, and *c’ = (c’_0_, c_1_)* be the output of the partial decryption protocol with target secret key *s_k_*. Then, $\frac{t}{q}\left( {c'}_{0}+{c'}_{1}\cdot s_{k} \right)=m+v_{fresh}+v_{pd}$with

$$\left\| v_{pd} \right\|\leq\frac{t}{q}B_{smg}z$$

Where *B_smg_* is the bound of the smudging distribution. We observe that the additional noise does not depend on the target secret key.

[1] Y. Lindell, “How to simulate it–a tutorial on the simulation proof technique,” in *Tutorials on the Foundations of Cryptography*. Springer, 2017, pp. 277–346.

[2] J. Fan and F. Vercauteren, “Somewhat Practical Fully Homomorphic Encryption.” *IACR Cryptology ePrint Archive*, vol. 2012, p. 144, 2012
